# Heart-Type Fatty Acid-Binding Protein (H-FABP) as a Candidate Adjunctive Biomarker for Immune Checkpoint Inhibitor-Related Cardiotoxicity: Linking Early Immune–Metabolic Myocardial Injury with Translational Cardio-Oncology

**DOI:** 10.3390/ijms27114842

**Published:** 2026-05-27

**Authors:** Vincenzo Quagliariello, Massimiliano Berretta, Fabrizio Maurea, Maria Laura Canale, Andrea Paccone, Irma Bisceglia, Andrea Tedeschi, Marino Scherillo, Jacopo Santagata, Stefano Oliva, Christian Cadeddu Dessalvi, Pietro Forte, Cristiana D’Ambrosio, Tiziana Di Matola, Domenico Gabrielli, Nicola Maurea

**Affiliations:** 1 Division of Cardiology, Istituto Nazionale Tumori-IRCCS-Fondazione G. Pascale, 80131 Napoli, Italy; andrea.paccone@istitutotumori.na.it (A.P.); jacopo.santagata@istitutotumori.na.it (J.S.); pietro.forte@istitutotumori.na.it (P.F.); n.maurea@istitutotumori.na.it (N.M.); 2 Department of Clinical and Experimental Medicine, University of Messina, 98122 Messina, Italy; berrettama@gmail.com; 3 Radiology Division, University of L’Aquila, 67100 L’Aquila, Italy; fabriziomaurea1998@gmail.com; 4 U.O.C. Cardiologia, Ospedale Versilia, 55041 Lido di Camaiore, Italy; marialaura.canale@uslnordovest.toscana.it; 5 Servizi Cardiologici Integrati, Dipartimento Cardio-Toraco-Vascolare, Azienda Ospedaliera San Camillo Forlanini, 00152 Roma, Italy; irmabisceglia@gmail.com; 6 Cardiology, “Guglielmo da Saliceto” Hospital, 29121 Piacenza, Italy; andrea.tedeschimd@gmail.com; 7 Cardiologia Interventistica e UTIC, A.O. San Pio, Presidio Ospedaliero Gaetano Rummo, 82100 Benevento, Italy; marino.scherillo@libero.it; 8 Cardio-Oncology Unit, IRCCS Istituto Tumori, “Giovanni Paolo II”, 70124 Bari, Italy; s.oliva@oncologico.bari.it; 9 Department of Medical Sciences and Public Health, University of Cagliari, 09124 Cagliari, Italy; cadedduc@unica.it; 10 Cardiology Division, “F. Veneziale”, Molise Regional Health Company (ASREM), 86170 Isernia, Italy; cristianadambrosio1@virgilio.it; 11 UOC Biochimica Clinica, AORN Ospedali dei Colli-Monaldi-Cotugno-CTO, 80131 Napoli, Italy; tizianadimatola7@gmail.com; 12 U.O.C. Cardiologia, Dipartimento Cardio-Toraco-Vascolare, Azienda Ospedaliera San Camillo Forlanini, Roma-Fondazione per il Tuo Cuore-Heart Care Foundation, 50121 Firenze, Italy; dgabrielli@scamilloforlanini.rm.it

**Keywords:** cardio-oncology, immune checkpoint inhibitors, myocarditis, H-FABP, troponin, early biomarkers, cardiotoxicity

## Abstract

Immune checkpoint inhibitors (ICIs) have transformed the therapeutic landscape of oncology but are increasingly associated with cardiovascular immune-related adverse events (irAEs), including myocarditis, heart failure, arrhythmias, and vascular complications. Among these, ICI-associated myocarditis represents the most severe manifestation, often characterized by high mortality and challenging early diagnosis. Detecting subclinical myocardial injury before irreversible cardiomyocyte necrosis occurs remains a major unmet need in contemporary cardio-oncology. This narrative expert review critically examines the biological rationale, preclinical evidence, and emerging clinical data supporting the potential role of heart-type fatty acid-binding protein (H-FABP) as an adjunctive biomarker of early immune-mediated myocardial injury during ICI therapy. H-FABP is a small cytosolic lipid chaperone abundantly expressed in cardiomyocytes and rapidly released into the circulation following subtle membrane destabilization and metabolic stress, frequently preceding detectable troponin elevation in other forms of myocardial injury. Experimental studies support a mechanistic association between H-FABP release, inflammasome activation, cytokine amplification, mitochondrial dysfunction, and immune–metabolic cardiomyocyte stress. Preliminary clinical observations further suggest that H-FABP elevations may occur during ICI treatment even in the absence of overt myocarditis or concomitant increases in high-sensitivity cardiac troponins (hs-cTns). Although H-FABP cannot replace hs-cTn, which remains the cornerstone biomarker for the diagnosis of clinically significant ICI-associated myocarditis, its rapid kinetics and sensitivity to early metabolic membrane injury support its potential role as an investigational adjunctive biomarker for early surveillance and risk stratification. This approach may be particularly relevant in patients receiving high-risk combination ICI regimens or in individuals with pre-existing cardiovascular disease. However, current evidence remains limited, and large prospective multicenter studies integrating H-FABP with hs-cTns, natriuretic peptides, cardiac magnetic resonance imaging, and clinical outcomes are required before routine clinical implementation can be considered.

## 1. Introduction

**Figure 1:**
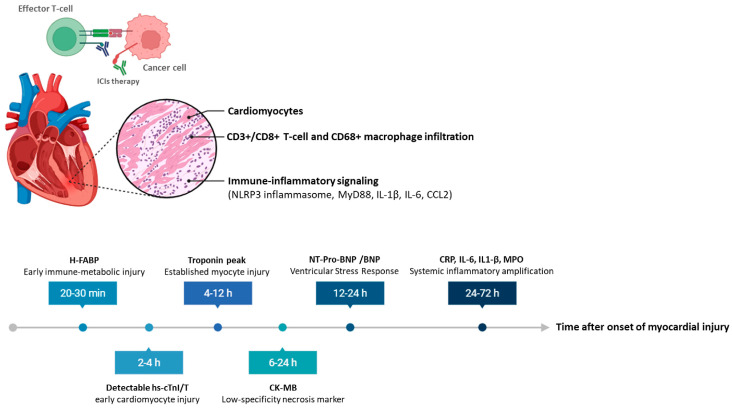
Conceptual mechanistic framework of immune–metabolic myocardial injury during immune checkpoint inhibitor therapy and proposed temporal kinetics of circulating cardiac biomarkers. The figure schematically illustrates the pathophysiological cascade linking immune checkpoint inhibitor (ICI) therapy to early myocardial injury and the hypothesized differential kinetics of circulating cardiac biomarkers. PD-1/programmed cell death protein-1, PD-L1/programmed death-ligand 1, and CTLA-4/cytotoxic T-lymphocyte-associated protein 4 blockade promote activation of CD3^+^/CD8^+^ effector T lymphocytes and infiltration of CD68^+^ pro-inflammatory macrophages into myocardial tissue, generating a characteristic inflammatory signature associated with ICI-mediated myocarditis. Within cardiomyocytes, immune activation triggers NLRP3 inflammasome signaling, MyD88-dependent inflammatory pathways, and release of IL-1β, IL-6, IL-8, CCL2, and other cytokines, leading to mitochondrial dysfunction, oxidative stress, impaired fatty-acid oxidation, and early sarcolemmal destabilization. The lower timeline represents a proposed conceptual sequence of biomarker release following immune-mediated cardiomyocyte injury. H-FABP, a small cytosolic lipid chaperone involved in intracellular fatty-acid trafficking, is hypothesized to rise during the earliest phase of immune–metabolic myocardial stress, potentially preceding overt structural injury. High-sensitivity cardiac troponins (hs-cTnI/T) become detectable later and reflect established cardiomyocyte injury and structural membrane disruption. CK-MB, natriuretic peptides (NT-proBNP/BNP), and systemic inflammatory biomarkers (CRP, IL-6, IL-1β, MPO) may subsequently increase as myocardial injury, ventricular stress, and systemic immune amplification progress. Importantly, the proposed biomarker kinetics illustrated in this figure are conceptual and derived from currently available evidence in acute myocardial injury, inflammatory cardiac disease, preclinical ICI models, and limited translational observations. These temporal dynamics have not yet been prospectively validated in patients with ICI-associated myocarditis and should therefore be interpreted as a hypothesis-generating translational framework rather than a definitive clinical model.

Immune checkpoint inhibitors (ICIs) have profoundly transformed the therapeutic landscape of modern oncology, significantly improving survival across a broad spectrum of solid tumors and hematologic malignancies, including melanoma, non-small-cell lung cancer, renal cell carcinoma, and Hodgkin lymphoma [1]. Parallel to their expanding clinical use, however, there has been growing recognition of cardiovascular immune-related adverse events (CV-irAEs), a heterogeneous group of toxicities that, although relatively uncommon, may be severe, rapidly progressive, and potentially fatal [2]. Contemporary pharmacovigilance studies, multicenter registries, and real-world cardio-oncology cohorts have shown that ICI-associated cardiovascular toxicity is likely underdiagnosed and may occur more frequently than initially appreciated, particularly in patients receiving combination immune checkpoint blockade or with pre-existing cardiovascular disease [3,4]. The clinical spectrum of ICI-associated cardiotoxicity includes asymptomatic biomarker elevation, myocarditis, heart failure, arrhythmias, pericardial disease, vasculitis, accelerated atherosclerosis, Takotsubo-like syndromes, and sudden cardiac death [5]. Among these manifestations, ICI-associated myocarditis remains the most feared complication because of its fulminant clinical course and disproportionately high mortality. Although the estimated incidence is relatively low, generally ranging between 0.3% and 1.5% in observational studies, multicenter real-world analyses and international registries consistently report mortality rates approaching 25–50%, particularly in severe presentations associated with ventricular arrhythmias, conduction disturbances, or cardiogenic shock [6,7,8]. Importantly, fatal events may occur even in patients with preserved left ventricular ejection fraction, emphasizing the need for heightened clinical vigilance and improved early detection strategies [9]. The pathophysiology of ICI-induced myocardial injury is complex and incompletely understood but appears to involve dysregulated immune activation, loss of peripheral tolerance, cross-reactivity between tumor and cardiac antigens, and amplification of pro-inflammatory cytokine cascades, ultimately resulting in immune-mediated myocyte injury, mitochondrial dysfunction, and myocardial fibrosis [10]. Histopathological studies of ICI myocarditis typically demonstrate dense infiltration by activated CD3^+^/CD8^+^ T lymphocytes and macrophages, accompanied by inflammatory cytokine release and myocyte necrosis [11]. Current surveillance strategies predominantly rely on high-sensitivity cardiac troponins (hs-cTns) and natriuretic peptides, biomarkers that are clinically validated and strongly associated with overt myocardial injury and adverse outcomes [12]. However, these markers primarily reflect established cardiomyocyte necrosis or hemodynamic stress rather than the earliest phases of immune-mediated metabolic dysfunction. Consequently, clinically silent or potentially reversible myocardial injury may remain undetected until structural damage has already developed [13]. Heart-type Fatty Acid-Binding Protein (H-FABP), a 15-kDa cytosolic lipid chaperone abundantly expressed in cardiomyocytes, has emerged as a potential investigational biomarker of early myocardial injury [14]. Due to its small molecular size, cytosolic localization, and rapid diffusion kinetics, H-FABP is released into the circulation within minutes of membrane destabilization or metabolic stress, often preceding detectable troponin elevation [15]. Beyond serving as a marker of necrosis, H-FABP may reflect early oxidative stress, mitochondrial dysfunction, impaired fatty-acid handling, and subtle sarcolemmal injury, processes increasingly recognized as central components of immune-mediated cardiotoxicity [16]. Preliminary translational and clinical observations suggest that H-FABP may complement hs-cTns and natriuretic peptides by identifying an earlier phase of immune–metabolic myocardial perturbation during ICI therapy, particularly in high-risk populations [17]. Nevertheless, current evidence remains limited and largely exploratory, and the precise clinical role of H-FABP in cardio-oncology surveillance has yet to be established. The objective of this narrative expert review is to critically appraise the current understanding of ICI-associated cardiotoxicity, discuss the biological and pathophysiological rationale supporting H-FABP as a candidate adjunctive biomarker, summarize the available preclinical and clinical evidence, and outline future research directions required to define its potential role in early diagnosis, risk stratification, and multimodal surveillance strategies within contemporary cardio-oncology (Figure 1).

## 2. Pathophysiological Basis of H-FABP Release in ICI-Associated Myocardial Injury

### 2.1. Cardiomyocyte Biology and Early Release Kinetics of H-FABP

Notably, H-FABP is a protein that functions as a pivotal intracellular lipid chaperone within cardiomyocytes. It binds long-chain fatty acids (LCFAs) with high affinity (Kd ≈ 1 µM) and shuttles them from the sarcolemma to mitochondrial and peroxisomal sites of β-oxidation, facilitating efficient energy production in the highly oxidative myocardium [9]. Under physiological conditions, H-FABP acts as a critical buffer in fatty-acid flux, maintaining lipid homeostasis, preventing cytotoxic accumulation of acyl-CoA intermediates, and supporting ATP generation necessary for continuous contraction [10]. Because nearly 60–70% of myocardial ATP derives from fatty-acid oxidation, this protein is integral to the metabolic fidelity of the heart. Unlike sarcomeric proteins such as cardiac troponins (which are largely structural and sequestered within the contractile apparatus), H-FABP resides freely in the cytosolic compartment and constitutes approximately 0.02% of total myocardial protein content [11]. This distinct localization explains its extremely rapid efflux following minimal sarcolemmal disruption.

Experimental kinetic studies demonstrate that H-FABP becomes detectable in peripheral blood within 20–30 min after myocardial injury, peaks at 6–8 h, and generally returns to baseline within 24 h, owing to its short plasma half-life (~20 min) and renal clearance through glomerular filtration [12]. By contrast, hs-cTns typically begin to rise 3–6 h post-injury and remain elevated for several days. This kinetic distinction delineates a critical temporal window of opportunity in which H-FABP signals incipient or reversible cardiomyocyte stress, preceding irreversible necrosis marked by troponin release [13]. Moreover, cellular changes sufficient to increase H-FABP need not involve frank lysis or apoptosis; subtle alterations in sarcolemmal phospholipid architecture, transient membrane blebbing, or early mitochondrial dysfunction can trigger diffusion of this small, soluble protein into the extracellular space [14,15,16]. Such phenomena occur in settings of oxidative stress, calcium overload, or cytokine-mediated injury, all of which characterize the myocardial immune response to immune checkpoint inhibitors (ICIs). Indeed, ICIs unleash cytotoxic T-cell and macrophage activity that generates reactive oxygen and nitrogen species, interferon-γ, and pro-inflammatory cytokines, such as Interleukin (IL)-1β, IL-6 and Tumour Necrosis Factor (TNF)-α [17]. These mediators compromise mitochondrial membranes, reduce cardiolipin integrity, and promote lipid peroxidation, processes that weaken the sarcolemmal barrier without immediate myofibrillar destruction. The consequent micro-leakage of cytosolic components, most notably H-FABP, reflects metabolic stress and membrane instability preceding the phase of irreversible necrosis later captured by troponin release [17]. Furthermore, metabolic remodeling induced by checkpoint blockade shifts myocardial substrate preference away from fatty acids toward glucose and ketones, mirroring the “metabolic inflexibility” seen in inflammatory cardiomyopathies [17]. This maladaptation depletes intracellular pools of H-FABP-bound fatty acids and accentuates lipid droplet accumulation, amplifying oxidative stress; therefore, high circulating H-FABP may represent both a marker and mediator of impaired lipid utilization.

Increased extracellular concentrations correlate with heightened expression of fatty acid translocase (CD36) and carnitine palmitoyltransferase-1 (CPT-1) suppression, molecular hallmarks of mitochondrial energetic failure [18]. Hence, the biomarker embodies a biochemical fingerprint of disrupted β-oxidation and immune–metabolic coupling within cardiomyocytes exposed to ICIs. Clinically, these properties grant H-FABP unique predictive potential. Its fast kinetics and sensitivity to microinjury render it a plausible “first-responder” biomarker, capable of flagging myocardial distress during the earliest, reversible phase of ICI-associated cardiotoxicity, when prompt immunosuppressive or cardioprotective interventions may avert progression to myocarditis or overt heart failure [19]. Early H-FABP elevations, particularly when dissociated from troponin or natriuretic peptide rises, could therefore delineate a transitional phenotype of immune-mediated cardiomyocyte stress, bridging the asymptomatic biochemical phase and clinically manifest myocarditis [20]. From a translational standpoint, integrating H-FABP measurement into biomarker panels alongside hs-cTns and NT-proBNP could enhance temporal sensitivity and refine risk stratification in cardio-oncology surveillance.

Notably, the biochemical evidences correlating H-FABP as a predictive marker lies in its cytosolic abundance, lipid-chaperone function, rapid diffusion kinetics and deep linkage to metabolic integrity. Within the inflammatory and oxidative milieu induced by immune checkpoint blockade, these features enable H-FABP to serve as an early sentinel of myocardial stress, providing a crucial diagnostic lead time before irreversible structural injury ensues (Figure 1).

### 2.2. Immune Checkpoint Dysregulation and Immunometabolic Cardiac Injury

Immune checkpoint inhibitors (ICIs) targeting Programmed Cell Death Protein 1 (PD-1), Programmed Death-Ligand 1 (PD-L1), Cytotoxic T-Lymphocyte-Associated Protein 4 (CTLA-4), and OX40 (CD134/TNFRSF4) profoundly reshape immune homeostasis by removing inhibitory signals that normally restrain T-cell activation [21]. While this unleashes robust antitumor cytotoxicity, it simultaneously reduces immune peripheral tolerance and exposes the myocardium to a form of immune-mediated injury that resembles accelerated autoimmune myocarditis [22]. Loss of PD-1 signaling heightens T-cell receptor responsiveness and promotes sustained PI3K/Akt activation, Interferon-γ production and expansion of highly cytotoxic CD8^+^ effector populations, many of which infiltrate cardiac tissue in both human and preclinical models. Moreover, CTLA-4 blockade intensifies co-stimulatory signaling through CD28, further amplifying clonal expansion and cytokine release [23]. These immunologic alterations create a microenvironment in which cardiomyocytes become vulnerable targets through mechanisms that include molecular mimicry between tumor and cardiac antigens, bystander activation of polyclonal T cells in the presence of high cytokine loads, and CCR2-dependent recruitment of inflammatory macrophages that perpetuate tissue damage through secretion of TNF-α, IL-1β, IL-6, and reactive oxygen species [24] (Table 1). In human cardiomyocytes, the transition from immune activation to overt myocardial injury is critically mediated by progressive immunometabolic dysregulation. One of the earliest and most biologically relevant events is activation of the NLRP3 inflammasome, a cytosolic danger-sensing platform highly responsive to mitochondrial oxidative stress, intracellular calcium perturbation, oxidized phospholipids, ATP depletion, and reactive oxygen species generated during immune engagement [25]. Upon activation, NLRP3 recruits ASC and caspase-1, enabling cleavage and maturation of pro-IL-1β and pro-IL-18. These cytokines exert several metabolic effects, such as: IL-1β impairs mitochondrial oxidative phosphorylation, destabilizes complex I activity, and increases ROS generation; IL-18 enhances IFN-γ production by T and NK cells, intensifying cytotoxic pressure on myocytes; IL-6 shifts substrate preference away from mitochondrial fatty-acid oxidation toward glycolysis, weakening energetic efficiency in a tissue heavily reliant on β-oxidation. Moreover, high TNF-α levels destabilizes the sarcolemmal lipid bilayer by promoting sphingomyelinase activation, ceramide accumulation, and lipid peroxidation [26,27]. The cumulative effect of these pathways is the development of a highly pro-oxidative and energetically compromised intracellular milieu characterized by mitochondrial swelling, impaired mitophagy, disruption of fatty-acid trafficking, loss of membrane phospholipid integrity, and increased sarcolemmal fragility. Importantly, this phase may occur before irreversible sarcomeric disruption or overt cardiomyocyte necrosis becomes established.

Within this hostile environment, cardiomyocytes undergo subtle but consequential membrane destabilization that precedes overt myofibrillar destruction. Because H-FABP is a small, highly soluble cytosolic lipid chaperone intimately involved in intracellular fatty-acid transport, even minimal disruptions in membrane integrity, on the order of transient blebbing, oxidative phospholipid damage, or early mitochondrial dysfunction, suffice to permit its rapid efflux into the extracellular space [28]. Experimental evidence suggests that oxidative stress, cytokine exposure, and direct immune-cell interaction induce nanoscale sarcolemmal defects and reversible membrane permeability alterations that facilitate early leakage of cytosolic proteins long before structural biomarkers such as cardiac troponins become detectable [28]. Therefore, H-FABP release appears to reflect early immune-mediated membrane perturbation and metabolic stress rather than irreversible cardiomyocyte destruction. Importantly, metabolic reprogramming during ICI exposure, marked by downregulation of Peroxisome proliferator-activated receptor alpha (PPAR-α), CPT-1 suppression, and accumulation of dysfunctional mitochondria, increases reliance on H-FABP-mediated lipid buffering and exacerbates intracellular fatty-acid toxicity [29]. Under such conditions, the cytosolic pool of H-FABP becomes more prone to leakage, linking its extracellular appearance not merely to cell injury but to the broader collapse of cardiomyocyte metabolic homeostasis (Table 1). Preclinical ICI models consistently reproduce this integrated immune–metabolic phenotype. Murine and in vitro studies employing dual PD-1/CTLA-4 blockade demonstrate synchronous increases in H-FABP and pro-inflammatory cytokines, along with NLRP3 activation and mitochondrial oxidative derangements [30,31]. In these models, H-FABP elevation reliably precedes troponin release, rises in NT-proBNP, or structural imaging abnormalities, suggesting that it is temporally coupled to the earliest phases of immune-mediated metabolic compromise rather than to the downstream necrotic cascade. Taken together, these mechanistic observations support the concept that H-FABP may identify a biologically early and potentially reversible phase of ICI-associated myocardial injury, characterized predominantly by immune-driven metabolic stress and sarcolemmal instability rather than overt structural necrosis. This alignment across mechanistic biology, metabolic dysfunction, and biomarker kinetics positions H-FABP as a highly plausible early sentinel of ICI-associated myocardial stress, one that reflects a reversible window of injury in which prompt therapeutic intervention may prevent transition to fulminant myocarditis or chronic cardiomyopathy (Table 1). Collectively, these mechanisms provide the biological rationale for the early release of H-FABP during immune-mediated cardiomyocyte stress and support its investigation as a candidate adjunctive biomarker in cardio-oncology.

### 2.3. Preclinical Evidence Supporting Early H-FABP Release

#### 2.3.1. Human Cardio-Immune Co-Culture Models

Experimental models employing human cardiomyocytes co-cultured with activated immune cells have provided a highly instructive window into the pathobiology of ICI-associated myocardial injury [32,33,34]. When cardiomyocytes are exposed to lymphocytes rendered hyperactive through dual PD-1 and CTLA-4 blockade, they undergo a remarkably rapid and coordinated metabolic and inflammatory collapse that mirrors key features of ICI myocarditis in vivo. In these models, blockade of Programmed Cell Death Protein 1 (PD-1) removes inhibitory phosphorylation brakes on T-cell receptor (TCR) signaling, sustaining Zeta-chain-associated protein kinase 70 (ZAP70), phosphoinositide 3-kinase/protein kinase B (PI3K–Akt), and nuclear factor kappa-light-chain-enhancer of activated B cells (NF-κB) activity, while CTLA-4 inhibition amplifies co-stimulatory CD28–B7 engagement, promoting full effector T-cell differentiation [35,36]. The resulting immune cells adopt a cytotoxic, pro-inflammatory phenotype characterized by abundant IFN-γ, granzyme B, perforin, and TNF-α secretion, conditions that accelerate cardiomyocyte stress even in the absence of overt antigen specificity. Within this inflammatory microenvironment, cardiomyocytes exhibit prompt activation of the NLRP3 inflammasome, a central platform integrating diverse cellular danger signals [37] (Table 2). Loss of mitochondrial membrane potential, increased generation of superoxide and hydroxyl radicals, oxidized mitochondrial DNA, and intracellular potassium efflux converge to trigger assembly of the NLRP3–ASC–caspase-1 complex. This activation results in maturation and release of IL-1β and IL-18, which further amplify the inflammatory loop by impairing mitochondrial oxidative phosphorylation, disturbing calcium homeostasis, and augmenting ROS production. IL-6 secretion, stimulated in part by both T cells and stressed cardiomyocytes, further drives metabolic reprogramming toward glycolysis and away from mitochondrial fatty-acid oxidation, worsening bioenergetic insufficiency [38]. As these pathways take hold, cardiomyocytes progressively lose their ability to maintain membrane integrity and lipid homeostasis, rendering them exquisitely vulnerable to immune-induced metabolic injury (Table 2). One of the most illuminating observations arising from these co-culture experiments is the synchronous release of H-FABP from cardiomyocytes under PD-1/CTLA-4 blockade. H-FABP efflux occurs in close temporal association with cytokine surges and inflammasome activation, often within the first hour of immune challenge [39]. Importantly, this release precedes any robust elevation of cardiac troponins, which require more extensive myofibrillar disruption and activation of necrotic or apoptotic cascades before entering the circulation. The early appearance of H-FABP in the extracellular milieu therefore reflects subtle but significant sarcolemmal destabilization, likely driven by lipid peroxidation, ROS-mediated phospholipid damage, and reversible mitochondrial permeability perturbations, all hallmark consequences of inflammasome-driven immune stress [40]. Because H-FABP is a small, freely soluble cytosolic protein, even nanometer-scale breaches in the plasma membrane permit its leakage, enabling it to function as a proximal biochemical reporter of early cellular distress long before structural collapse occurs. These findings establish a mechanistic continuum linking checkpoint blockade → T-cell hyperactivation → inflammasome engagement → mitochondrial dysfunction → early membrane injury → H-FABP release, providing a biologically coherent rationale for the biomarker’s predictive value in patients receiving ICIs.

Notably, the co-culture systems show that H-FABP elevation is not merely a byproduct of mechanical or hemodynamic strain but arises directly from immune-mediated metabolic perturbation, anchoring the biomarker firmly in the pathophysiology of ICI-induced myocardial injury [41]. The reproducibility of these observations across independent experimental models strengthens the case for H-FABP as a sensitive indicator of immune–metabolic instability within cardiomyocytes, a phase that may remain clinically silent but potentially reversible if detected at its inception. In translational terms, the precedence of H-FABP over troponin in these controlled immune-cardiac interactions underscores its potential utility as an early sentinel biomarker, capable of identifying the inflection point at which reversible immune-driven metabolic stress transitions toward irreversible myocyte death. This temporal advantage is precisely the diagnostic window needed to intervene with immunosuppression or treatment modification before fulminant myocarditis or significant left ventricular dysfunction develops. By bridging molecular immunology with clinically interpretable biochemical signaling, these co-culture studies offer a compelling, mechanistically grounded framework for incorporating H-FABP into modern cardio-oncology surveillance strategies [42] (Table 2).

#### 2.3.2. Analytical Methodology and Standardization of H-FABP Assessment

Importantly, H-FABP can be measured in serum or plasma using several analytical platforms, most commonly enzyme-linked immunosorbent assays (ELISA), immunoturbidimetric assays, chemiluminescent immunoassays, and rapid point-of-care immunochromatographic tests. Among these, ELISA-based methods are widely used in translational and experimental studies because of their analytical sensitivity and suitability for batch processing, whereas immunoturbidimetric and automated immunoassay platforms may be more appropriate for clinical laboratories requiring shorter turnaround times and higher throughput [43]. Point-of-care assays provide rapid qualitative or semi-quantitative results and may be attractive in emergency settings, but their limited analytical precision and inter-platform variability currently restrict their use in biomarker-driven cardio-oncology trials. For future prospective studies in patients treated with immune checkpoint inhibitors, assay selection should be guided by analytical performance, reproducibility, lower limit of detection, intra- and inter-assay coefficients of variation, dynamic range, and compatibility with serial sampling. Because H-FABP is a low-molecular-weight cytosolic protein with rapid release and clearance kinetics, timing of blood collection is particularly relevant. Sampling strategies should ideally include a pre-treatment baseline value, early on-treatment measurements during the highest-risk period for ICI-associated myocarditis, and repeated assessments in the presence of symptoms, electrocardiographic abnormalities, troponin changes, or systemic immune-related adverse events [44]. Static single-threshold interpretation is unlikely to be sufficient in this setting; therefore, patient-specific changes from baseline may be more informative than isolated absolute values. Pre-analytical conditions also require strict harmonization. Serum or plasma matrix, anticoagulant type, processing delay, centrifugation protocol, storage temperature, freeze–thaw cycles, and sample hemolysis may all influence measured H-FABP concentrations. These aspects are particularly important in multicenter studies, where variability in sample handling may obscure small but clinically meaningful biomarker changes. Standard operating procedures should therefore define blood collection tubes, maximum time to processing, storage conditions, allowable freeze–thaw cycles, and centralized versus local laboratory analysis [45]. Another major methodological issue is renal function. H-FABP is cleared predominantly through glomerular filtration, and reduced estimated glomerular filtration rate may increase baseline circulating concentrations independently of acute myocardial injury. This is especially relevant in oncology patients who may have chronic kidney disease, nephrotoxic treatment exposure, dehydration, or ICI-related nephritis. Future studies should therefore incorporate renal function-adjusted interpretation, stratified analyses according to estimated glomerular filtration rate, and preferably delta-based thresholds rather than universal absolute cut-offs [46]. From a trial-design perspective, H-FABP should not be evaluated as an isolated diagnostic test but as part of a multimodal biomarker strategy. Future protocols should predefine absolute and percentage changes in H-FABP, establish reference change values, and adjudicate biomarker trajectories against clinically meaningful endpoints such as high-sensitivity cardiac troponin elevation, cardiac magnetic resonance findings, myocarditis diagnosis, arrhythmic events, heart failure, treatment interruption, and major adverse cardiovascular events. Ideally, H-FABP measurements should be integrated with hs-cTns, NT-proBNP, inflammatory markers, ECG, echocardiography, and cardiac magnetic resonance imaging. Such standardization will be essential to determine whether H-FABP provides incremental diagnostic or prognostic value beyond established biomarkers and whether it can move from an exploratory signal to actionable decision support in cardio-oncology. Because H-FABP undergoes predominant renal clearance through glomerular filtration, impaired renal function may significantly increase baseline circulating concentrations independently of acute myocardial injury. This issue is particularly relevant in oncology patients, in whom chronic kidney disease, treatment-related nephrotoxicity, dehydration, or ICI-associated nephritis are frequently encountered. Consequently, interpretation of H-FABP should preferentially rely on serial delta changes integrated with renal function assessment rather than isolated absolute thresholds.

### 2.4. Clinical and Translational Evidence of H-FABP in ICI Cardiotoxicity

The clinical investigation of H-FABP during ICI-mediated cardiotoxicity is still in its early stages, yet the available human data already offer compelling insight into its potential role as a sensitive indicator of subclinical myocardial stress (Table 3). The most informative evidence to date arises from a prospective cohort study in which serial measurements of H-FABP were performed in cancer patients receiving PD-1, PD-L1, or CTLA-4 inhibitors [47]. In this study, circulating H-FABP levels increased significantly at both 3 and 6 months compared with baseline, even though high-sensitivity cardiac troponins (hs-cTns) and natriuretic peptides (NT-proBNP) remained stable throughout follow-up. Importantly, these elevations were not associated with clinically overt myocarditis or symptomatic cardiac events, suggesting that H-FABP may be detecting a biochemical intermediate state of immune-mediated cardiomyocyte stress, one that precedes the structural injury required to release troponins or compromise ventricular function [48]. This discordance between H-FABP and traditional cardiac biomarkers is particularly revealing. Troponins, due to their structural anchoring within the contractile apparatus, mark overt myocyte necrosis or irreversible membrane rupture, whereas H-FABP, with its cytosolic distribution and rapid diffusibility, appears to flag earlier, metabolically driven microinjury [49,50,51] (Table 3). The fact that H-FABP rises independently of troponin reinforces its unique kinetic niche and suggests that cumulative exposure to ICIs, even in the absence of fulminant myocarditis, may impose sustained immunometabolic strain on cardiomyocytes. This phenomenon aligns closely with preclinical observations in which dual checkpoint blockade consistently triggers a coordinated surge in H-FABP, IL-1β, and IL-6, alongside NT-proBNP elevations and NLRP3 inflammasome activation [52,53]. Taken together, these observations outline a consistent biological framework in which H-FABP emerges as an early marker of immune-mediated metabolic stress during ICI treatment. Additional signals from translational reports, registry analyses, and small observational cohorts reinforce this pattern, with H-FABP elevations appearing most frequently in patients receiving combination checkpoint blockade, regimens associated with the greatest risk of myocarditis. Collectively, these data suggest that H-FABP reflects the intensity of immune pressure on cardiomyocytes rather than representing a nonspecific byproduct of systemic stress (Table 3).

## 3. Comparative Biomarkers and the Rationale for Integrating H-FABP into Modern Cardio-Oncology Surveillance

The current diagnostic architecture for suspected ICI myocarditis remains centered on hs-cTns, supported by consensus recommendations from the International Cardio-Oncology Society (IC-OS) and corroborated by observational registries demonstrating its high sensitivity for immune-mediated myocardial injury [54,55]. Natriuretic peptides, while less specific, complement troponins by reflecting ventricular wall stress and hemodynamic load [56]. However, neither biomarker reliably detects the earliest stages of immune-driven metabolic injury, particularly in patients who remain asymptomatic or present with vague, non-cardiac complaints [57,58]. Within this framework, H-FABP offers a distinctive advantage due to its rapid release kinetics and heightened sensitivity to sublethal membrane destabilization. Its incorporation into surveillance protocols holds particular promise in scenarios where clinical suspicion is elevated yet troponin remains within normal limits, a situation commonly encountered early in the ICI treatment course [59]. A pragmatic monitoring strategy would involve obtaining baseline ECG and echocardiographic parameters coupled with hs-cTns and, where available, H-FABP. Subsequent cycle-by-cycle assessment during the initial 6–8 weeks, generally considered the period of highest myocarditis risk, would allow clinicians to detect subtle deviations from biochemical baseline [60]. If H-FABP rises in isolation, without concurrent troponin elevation, this pattern may indicate incipient immunometabolic stress, prompting intensified monitoring, earlier cardiology involvement, or expedited cardiac MRI with T1/T2 mapping [61]. Should H-FABP and hs-cTns rise concurrently, the likelihood of active ICI myocarditis increases substantially, warranting swift diagnostic clarification and immediate initiation of immunosuppression. In this integrated model, H-FABP does not compete with troponin but rather complements it, offering a temporal lead that may be essential for preventing irreversible cardiomyocyte loss and ensuring continuity of potentially life-prolonging cancer therapy.

## 4. Current Clinical Limitations and the Path Toward Rigorous Validation

Despite encouraging preliminary findings, several limitations constrain immediate clinical implementation of H-FABP as a routine biomarker in ICI surveillance. To date, no large-scale prospective cohort has validated H-FABP against CMR-guided adjudication of myocarditis, the gold standard for non-invasive diagnosis in cardio-oncology. The absence of trials linking H-FABP dynamics to hard cardiovascular outcomes, such as MACEs, arrhythmic events, or hospitalization, limits our ability to define clinically meaningful thresholds or interpret isolated elevations confidently [62,63]. Moreover, inter-assay variability and susceptibility to renal clearance effects complicate universal cut-off determination, especially in oncology populations where renal dysfunction is prevalent [64]. Although the delta-change (absolute or percentage increase over baseline) may ultimately prove more informative than static thresholds, its diagnostic fidelity has yet to be systematically evaluated in ICI-treated cohorts. Finally, current datasets are underpowered to determine the incremental value of H-FABP over hs-cTns, making it essential to conduct larger, mechanistically anchored studies capable of quantifying whether early detection meaningfully improves patient outcomes [65]. In light of these gaps, the translational trajectory of H-FABP will depend on well-designed prospective studies integrating serial biomarker sampling, standardized imaging endpoints, systematic clinical adjudication, and stratification according to ICI regimen and baseline cardiovascular risk. If such investigations confirm that H-FABP identifies a reversible, immune–metabolic phase of myocardial injury before structural damage occurs, the biomarker could become a foundational component of next-generation cardio-oncology surveillance, offering crucial lead-time for intervention and preserving access to lifesaving immunotherapy [66,67].

## 5. Ongoing Projects and Trial Landscape Relevant to Biomarker-Guided Care

Although H-FABP itself is not yet the focus of an interventional trial in ICI cardiotoxicity, the biomarker-guided paradigm is advancing (Table 4). The STRICT study (NCT06337097) is evaluating whether troponin-based monitoring in ICI-treated patients improves outcomes, reflecting the community’s move toward structured surveillance that could readily accommodate fast-kinetic markers such as H-FABP in future iterations [68]. Parallel efforts include abatacept dosing trials for established ICI myocarditis (NCT05195645) and multicenter registries characterizing ICI-associated myocarditis phenotypes and outcomes (NCT04294771), which provide platforms where exploratory biomarkers, including H-FABP, can be embedded and benchmarked against imaging and clinical endpoints [69,70]. Additionally, genomic studies of susceptibility to ICI myocarditis (NCT06734689) may delineate risk strata in which early injury signals like H-FABP have maximal predictive yield [71]. A further observational project (NCT06309862) explicitly aims to identify early myocardial changes with circulating biomarkers in ICI-treated patients, conceptually aligning with H-FABP’s use case even if not named a priori [72]. Collectively, these ongoing initiatives illustrate the progressive transition from conventional troponin-centered monitoring toward integrated multimarker surveillance strategies capable of capturing earlier and potentially reversible phases of immune-mediated myocardial injury. Within this evolving framework, H-FABP may eventually be evaluated as part of mechanism-oriented biomarker panels combining hs-cTns, natriuretic peptides, inflammatory mediators, and advanced imaging modalities. Future prospective investigations should therefore focus on determining whether incorporation of H-FABP into structured surveillance algorithms improves the early identification of clinically relevant cardiotoxicity, facilitates more timely cardiac imaging or immunosuppressive intervention, and ultimately reduces interruption of potentially life-prolonging immunotherapy. Particular clinical interest may reside in high-risk populations, including patients receiving dual immune checkpoint blockade and individuals with pre-existing cardiovascular disease, in whom earlier recognition of immune–metabolic myocardial stress may have the greatest impact on outcomes. If validated in adequately powered multicenter cohorts, H-FABP could emerge as a clinically useful adjunctive biomarker within future consensus-based cardio-oncology surveillance pathways (Table 4).

## 6. Discussion

**Figure 2:**
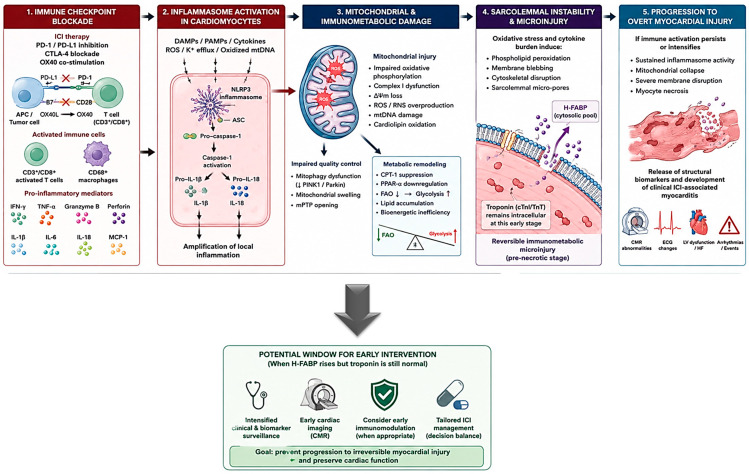
Proposed immunometabolic cascade underlying immune checkpoint inhibitor (ICI)-associated myocardial injury and early H-FABP release. The figure illustrates a conceptual mechanistic framework linking PD-1/PD-L1 and CTLA-4 blockade to immune-mediated myocardial injury. Activated CD3^+^/CD8^+^ T cells and CD68^+^ macrophages promote cytokine release, NLRP3 inflammasome activation, oxidative stress, and mitochondrial dysfunction within cardiomyocytes, leading to metabolic remodeling, impaired fatty-acid oxidation, and early sarcolemmal destabilization. These processes may facilitate rapid cytosolic release of H-FABP before overt structural necrosis and significant troponin elevation occur. The lower panel summarizes the proposed temporal kinetics of circulating cardiac biomarkers during ICI-associated myocardial injury. This figure represents a conceptual translational model derived from current experimental and emerging clinical evidence and does not reflect prospectively validated biomarker kinetics in ICI myocarditis.

In the present review, we provide a clinically oriented synthesis of the biological and translational rationale supporting H-FABP as a candidate adjunctive biomarker of ICI-related myocardial injury (Figure 2). The central concept emerging from the currently available evidence is that ICI cardiotoxicity should not necessarily be interpreted exclusively as a late necrotic event identifiable only when troponin becomes abnormal or left ventricular dysfunction is evident. Rather, preclinical and early translational observations suggest that myocardial injury may evolve through an earlier phase of immune–metabolic cardiomyocyte stress, characterized by cytokine activation, mitochondrial dysfunction, oxidative lipid injury, and subtle sarcolemmal instability [15,24,25]. In this specific temporal window, H-FABP may provide biologically relevant information because of its rapid cytosolic release and close relationship with fatty-acid handling and membrane integrity [12,14]. From a clinical perspective, this distinction may be particularly relevant. ICI myocarditis is uncommon but frequently severe, often presenting early after treatment initiation and occasionally with preserved left ventricular ejection fraction [3,4]. In real-world cardio-oncology practice, clinicians are frequently confronted with diagnostically ambiguous scenarios, including mild symptoms, nonspecific ECG changes, isolated biomarker fluctuations, concomitant systemic immune-related adverse events, or cancer-related fatigue that may obscure early cardiac involvement. In these settings, reliance on troponin alone may theoretically identify some patients only after structural myocyte injury has already occurred [5]. Accordingly, H-FABP has been proposed as a potential marker of earlier immune–metabolic myocardial perturbation; however, this hypothesis remains insufficiently validated in prospective clinical cohorts. Importantly, H-FABP should not be viewed as a replacement for high-sensitivity cardiac troponin. Troponin remains the cornerstone biomarker for diagnosing clinically relevant myocardial injury and suspected ICI myocarditis [54,55]. The potential value of H-FABP lies instead in its possible complementary role within multimodal surveillance strategies (Figure 2). A practical interpretation model may involve three biomarker patterns. First, stable H-FABP and stable hs-cTns would support continuation of standard surveillance in clinically low-risk patients. Second, isolated H-FABP elevation with normal hs-cTn may suggest early immune–metabolic stress and justify intensified monitoring, repeat biomarker testing, ECG reassessment, and consideration of echocardiography or CMR in selected high-risk patients [59,60]. Third, concurrent elevation of H-FABP and hs-cTns should raise stronger suspicion for active myocardial injury and prompt urgent cardio-oncology evaluation, particularly when accompanied by symptoms, ECG abnormalities, arrhythmias, or rising natriuretic peptides [61]. Nevertheless, these proposed interpretative frameworks remain hypothetical and should currently be regarded as exploratory translational concepts rather than clinically validated algorithms. No prospective trial has yet demonstrated that H-FABP-guided surveillance improves diagnostic accuracy, reduces myocarditis-related morbidity, or alters clinical outcomes in patients receiving ICIs (Figure 2). The potential clinical utility of H-FABP may be greatest in selected high-risk populations. These include patients receiving combination ICI therapy, those with pre-existing cardiovascular disease, prior exposure to anthracyclines or thoracic radiotherapy, older patients, individuals with renal impairment requiring careful delta-based interpretation, and patients developing concurrent systemic irAEs. In these groups, a fast-kinetic biomarker could theoretically provide an earlier signal of myocardial vulnerability and help distinguish patients requiring closer follow-up from those suitable for routine monitoring. This approach is consistent with the broader evolution of cardio-oncology from reactive management of overt cardiotoxicity toward proactive surveillance and prevention [57,58]. A clinically meaningful H-FABP strategy would likely require serial rather than isolated measurement. Because H-FABP is rapidly cleared and influenced by renal function, absolute thresholds may be less informative than patient-specific changes from baseline [12,46]. Future protocols should therefore prioritize baseline assessment before ICI initiation, early cycle-based sampling during the first 6–12 weeks, and predefined absolute and percentage delta changes. Such interpretation should always be integrated with hs-cTns, NT-proBNP, ECG, symptoms, renal function, inflammatory markers, and imaging findings (Figure 2). In this sense, H-FABP is best conceptualized as one component of a multimodal diagnostic pathway rather than a standalone decision-making tool [60,61]. An important limitation relates to the incomplete cardiac specificity of H-FABP. Although predominantly expressed in cardiomyocytes, H-FABP may also be released under conditions of systemic inflammation, skeletal muscle injury, severe metabolic stress, sepsis, or cachexia, all of which are relatively common in oncology populations. In addition, overlap syndromes involving ICI-related myocarditis and myositis are increasingly recognized in clinical practice and may complicate interpretation of circulating muscle-associated biomarkers. In these settings, isolated H-FABP elevation cannot be considered diagnostic of myocardial inflammation and should always be interpreted in conjunction with high-sensitivity troponins, electrocardiography, cardiac imaging, inflammatory markers, clinical presentation, and multidisciplinary cardio-oncology assessment. An additional challenge relates to biomarker specificity. H-FABP is not exclusively cardiac-specific and may increase in several non-cardiac conditions frequently encountered in oncology patients, including renal dysfunction, systemic inflammation, skeletal muscle injury, sepsis, and ICI-related myositis. This is particularly important because overlap syndromes involving myocarditis and myositis are increasingly recognized during ICI therapy and may complicate interpretation of circulating muscle-related biomarkers. Consequently, isolated H-FABP elevation should be interpreted cautiously and always within the broader clinical, laboratory, and imaging context. Several limitations currently prevent routine implementation. First, available clinical data remain limited and largely exploratory [47]. Second, no universally accepted cut-off exists for ICI-treated patients. Third, assay variability and renal clearance may affect reproducibility [46,64]. Fourth, isolated H-FABP elevation may reflect nonspecific myocardial stress rather than definite myocarditis. Finally, it remains unknown whether H-FABP-guided monitoring improves hard clinical outcomes, reduces myocarditis-related mortality, prevents heart failure, or preserves oncologic treatment continuity [62,63,64,65]. These gaps underscore the need for prospective multicenter studies with standardized assays, serial biomarker sampling, CMR adjudication, and clinically relevant endpoints. The most promising future direction is the development of risk-adapted biomarker algorithms. In such models, H-FABP could potentially be used to identify very early myocardial stress, hs-cTns to confirm structural injury, NT-proBNP to define hemodynamic involvement, and CMR to characterize inflammation, edema, and fibrosis [61]. This layered approach could improve diagnostic confidence and help guide difficult clinical decisions regarding ICI interruption, corticosteroid initiation, escalation of immunosuppression, and safe rechallenge. However, whether earlier biomarker detection ultimately translates into improved cardiovascular or oncologic outcomes remains unknown and will require prospective validation in adequately powered studies. Ultimately, the goal is not only to detect myocarditis earlier but also to preserve access to life-prolonging immunotherapy whenever safely possible [63,66,67]. In conclusion, H-FABP represents a biologically plausible and clinically interesting investigational adjunctive biomarker for early ICI-related myocardial injury. Its rapid kinetics, cytosolic localization, and relationship with immune–metabolic cardiomyocyte stress support its potential role as an early warning signal before overt necrosis develops [12,15]. At present, however, available evidence should be considered hypothesis-generating rather than practice-changing. Its use should therefore remain investigational until validated in prospective studies. If future evidence confirms incremental diagnostic or prognostic value beyond established biomarkers, H-FABP may eventually become part of next-generation cardio-oncology surveillance pathways, particularly for patients at elevated risk of ICI myocarditis.

## 7. Methods

This manuscript was designed as a narrative expert review aimed at critically appraising the current biological, translational, and clinical evidence regarding immune checkpoint inhibitor (ICI)-associated cardiotoxicity and the emerging role of heart-type fatty acid-binding protein (H-FABP) as an adjunctive biomarker of early myocardial injury. Although the review was not conducted as a formal systematic review or meta-analysis, a structured literature search strategy was implemented to improve transparency, reproducibility, and comprehensiveness of study selection. A literature search was performed using the MEDLINE (PubMed), EMBASE, and Web of Science databases to identify relevant publications addressing ICI-associated cardiovascular toxicity, immune-mediated myocardial injury, cardiac biomarkers, and H-FABP biology. The search covered the period from January 2010 to April 2026 in order to capture both foundational and contemporary evidence in the rapidly evolving field of cardio-oncology. The search strategy combined Medical Subject Headings (MeSH) and free-text terms related to immune checkpoint inhibitors, myocarditis, cardiotoxicity, H-FABP, cardiac troponins, natriuretic peptides, inflammasome activation, mitochondrial dysfunction, and immune–metabolic myocardial injury. Boolean operators (“AND”, “OR”) were applied to optimize retrieval of relevant articles. The principal search strings used across the databases are summarized in Table 5.

Studies were considered eligible if they:

(i) were published in English with an available abstract;
(ii) reported original clinical, translational, or preclinical data;
(iii) investigated ICI-associated cardiotoxicity, immune-mediated myocardial injury, cardiac biomarkers, or related mechanistic pathways;
(iv) provided clinically or biologically relevant evidence regarding H-FABP kinetics, myocardial stress, inflammasome activation, oxidative injury, or mitochondrial dysfunction.

Both clinical and experimental studies were included in order to provide an integrated translational perspective bridging molecular mechanisms and cardio-oncology practice. Relevant observational studies, cohort analyses, translational investigations, and mechanistic experimental models were preferentially prioritized. Particular emphasis was placed on contemporary studies investigating biomarker-guided surveillance, immunometabolic myocardial injury, inflammasome signaling, and early detection strategies in ICI-treated patients. Therefore, the final selection of studies was qualitatively synthesized to provide a clinically oriented and mechanistically grounded overview of the potential role of H-FABP as an investigational adjunctive biomarker in ICI-associated myocardial injury. The final database access was performed on 27 April 2026.

## 8. Conclusions

In conclusion, H-FABP represents a biologically plausible and clinically attractive biomarker for early detection of ICI-related myocardial injury. Preliminary evidence indicates that it may rise earlier than troponin, providing a potential “lead-time advantage” in high-risk patients. Importantly, potential confounding factors and limited cardiac specificity currently preclude the use of H-FABP as a standalone diagnostic biomarker in routine clinical practice. While promising, its clinical adoption requires standardization, validation against hard outcomes, and integration into multimodal diagnostic strategies. Future research should aim to establish its role within comprehensive cardio-oncology surveillance frameworks.

## Figures and Tables

**Table 1:** Proposed immune–metabolic mechanisms linking immune checkpoint inhibition to early H-FABP release. Mechanistic pathways summarized in this table integrate established experimental findings with hypothesis-generating translational interpretations derived from preclinical and early clinical evidence.

Pathway	Immune Checkpoint Blockade Effect	Cardiomyocyte Metabolic & Structural Consequences	Resulting H-FABP Dynamics	Clinical and Translational Implications
Loss of PD-1 inhibitory signaling	Hyperactivation of CD8^+^ T cells; sustained TCR–ZAP70–PI3K/Akt activation; increased IFN-γ, granzyme B, perforin	Early immune pressure; mitochondrial stress; increased ROS; destabilization of membrane phospholipids	Early H-FABP release potentially preceding overt structural injury	Suggests cytotoxic immune engagement may trigger pre-necrotic cardiomyocyte stress detectable by H-FABP
CTLA-4 blockade and amplified CD28 co-stimulation	Increased clonal expansion; heightened TNF-α and IL-2 signaling; amplified pro-inflammatory milieu	Increased cytokine burden; impaired mitochondrial respiration; early sarcolemmal fragility	H-FABP may increase even in the absence of detectable troponin elevation	Reinforces the hypothesis that H-FABP may reflect combined immunologic and metabolic overload
Molecular mimicry & bystander activation	Cross-reactive T-cell responses; antigen-independent activation driven by cytokine storm	Diffuse myocardial involvement; oxidative and metabolic stress without overt necrosis	H-FABP elevation may occur before detectable CMR abnormalities in early phases	Supports the potential role of H-FABP in identifying diffuse microinjury even without focal myocarditis
CCR2-dependent macrophage recruitment	Monocyte/macrophage influx; secretion of TNF-α, IL-1β, IL-6, ROS	Lipid peroxidation; mitochondrial swelling; impaired Ca^2+^ handling	Potential increase in H-FABP release associated with membrane destabilization	Suggests cytokine-rich microenvironments may amplify immunometabolic vulnerability
NLRP3 inflammasome activation	ASC–caspase-1 assembly; conversion of pro–IL-1β/pro–IL-18 to active forms	Disruption of oxidative phosphorylation; increased ROS/RNS; impaired mitophagy	Experimental models suggest temporal association between NLRP3 activation and H-FABP release	Supports a putative link between inflammasome signaling and early biomarker release
Cytokine effects (IL-1β, IL-6, IL-18, TNF-α)	Enhanced inflammatory signaling; JAK/STAT3 activation; shift toward glycolytic metabolism	Bioenergetic failure; suppression of PPAR-α/CPT-1; mitochondrial dysfunction; lipid accumulation	H-FABP elevation may reflect impaired fatty-acid handling and metabolic buffering	Positions H-FABP as a potential indicator of metabolic maladaptation and altered fatty-acid oxidation
Oxidative stress & lipid peroxidation	ROS surge from immune–mitochondrial interactions	Phospholipid oxidation; membrane blebbing; reversible sarcolemmal injury	Early H-FABP increase has been observed in experimental models before detectable troponin elevation	Highlights the possible temporal advantage of H-FABP relative to structural myocardial biomarkers
Mitochondrial dysfunction & impaired mitophagy	PINK1/Parkin downregulation; mitochondrial permeability transition; ATP depletion	Increased vulnerability to immune-mediated injury; inability to maintain lipid homeostasis	H-FABP release may accompany mitochondrial and metabolic instability	Suggests H-FABP could identify potentially reversible phases of mitochondrial stress
Integrated immunometabolic phenotype (dual PD-1 + CTLA-4 blockade)	Synergistic immune activation; highest cytokine and ROS burden	Synchronous mitochondrial, metabolic, and membrane dysfunction	Experimental models have reported early H-FABP release patterns, whereas troponin elevation appears later	Provides a biologically plausible rationale for exploring H-FABP monitoring in high-risk combination ICI regimens

**Table 2:** Proposed preclinical mechanisms linking immune checkpoint blockade to early H-FABP release. Experimental findings summarized in this table integrate established preclinical evidence with hypothesis-generating translational interpretations regarding H-FABP dynamics during immune-mediated myocardial stress.

Preclinical Model	Key Immune and Molecular Events	Cardiomyocyte Metabolic & Structural Consequences	Biomarker Dynamics (H-FABP vs. Troponin)	Translational Implications
Human cardiomyocyte–T cell co-culture under dual PD-1 + CTLA-4 blockade	Hyperactivation of CD8^+^ T cells; sustained ZAP70–PI3K–Akt–NF-κB signaling; increased IFN-γ, TNF-α, granzyme B, perforin release; macrophage-like cytokine amplification	Rapid NLRP3 inflammasome activation; caspase-1–mediated maturation of IL-1β/IL-18; mitochondrial membrane depolarization; ROS and RNS surge; early sarcolemmal phospholipid peroxidation	Experimental models have reported early H-FABP release preceding detectable troponin elevation, potentially reflecting reversible membrane injury rather than overt necrosis	Suggests immunometabolic microinjury may occur during early ICI exposure and supports investigation of H-FABP as a candidate biomarker of pre-necrotic stress
Cytokine-primed cardiomyocyte models (IL-1β, IL-6 exposure)	Amplified inflammatory signaling; JAK/STAT3 activation; impaired mitochondrial fatty-acid oxidation; increased glycolytic shift	Bioenergetic collapse; oxidative phosphorylation impairment; lipid droplet accumulation; increased mitochondrial ROS and swelling	Elevated H-FABP has been associated with cytokine burden, whereas troponin may remain within normal range during early phases	Supports a potential relationship between inflammatory cytokine load and H-FABP release in immune-related myocardial stress
ROS-induction/oxidative injury models	Excessive superoxide/hydroxyl radical formation; mitochondrial DNA oxidation; disruption of cardiolipin-rich inner mitochondrial membrane	Loss of membrane potential; enhanced mitochondrial permeability transition; lipid peroxidation; reversible sarcolemmal microtears	H-FABP release has been observed early in oxidative stress models, whereas troponin elevation generally requires more extensive structural injury	Positions H-FABP as a potential upstream indicator of oxidative and metabolic myocardial stress
NLRP3 inflammasome genetic or pharmacologic modulation	Inhibition of NLRP3 or caspase-1 reduces IL-1β/IL-18 output and downstream immune activation	Mitigation of mitochondrial dysfunction and membrane instability; reduced ROS amplification	Experimental attenuation of inflammasome signaling has been associated with reduced H-FABP release	Supports a putative mechanistic relationship between inflammasome activation and early H-FABP dynamics
Dual ICI exposure (PD-1 + CTLA-4) in translational immuno-cardiac models	Synergistic increase in T-cell cytotoxicity; exaggerated TNF-α, IFN-γ, IL-6 signaling; immune–metabolic overload	Early mitochondrial swelling; impaired mitophagy; CPT-1 suppression; PPAR-α downregulation; metabolic inflexibility	Experimental studies suggest earlier H-FABP elevation patterns, whereas troponin increases appear more prominent during later injury phases	Provides a biologically plausible rationale for investigating H-FABP monitoring in high-risk combination ICI regimens
Assay performance and analytical considerations	NA	NA	ELISA and immunoturbidimetric assays demonstrate rapid analytical responsiveness; however, renal clearance and inter-assay variability may influence circulating concentrations	Highlights the need for assay harmonization and standardized ΔH-FABP thresholds before routine clinical implementation

**Table 3:** Clinical and translational evidence supporting H-FABP as a candidate biomarker in ICI-associated myocardial injury.

Study/Source	Study Design & Population	Biomarker Findings	Clinical Outcomes/Imaging Correlation
Yuan et al., 2021 [47]	Prospective single-center cohort; n = 19; mixed solid tumors (lung cancer, rectal cancer, breast cancer, renal cell carcinoma, melanoma, bladder cancer, gynecologic tumors); PD-1/PD-L1 inhibitors (nivolumab, sintilimab, durvalumab)	H-FABP increased significantly at 3 and 6 months, whereas cTnI and NT-proBNP remained stable	No clinical myocarditis, no major cardiovascular events, no significant LVEF decline; no CMR-guided adjudication
Quagliariello et al., 2024 [30]	Preclinical/translational cardio-immunology models; dual PD-1/CTLA-4 blockade	Increased H-FABP associated with IL-1β, IL-6, NT-proBNP elevation, and NLRP3 inflammasome activation	Early immune–metabolic myocardial alterations preceding overt structural injury
Observational cardio-oncology datasets [3,4,6]	Real-world ICI-treated populations; mixed solid tumors; predominantly PD-1/PD-L1 regimens	Sporadic H-FABP elevations, occasionally without matched troponin increase	Myocarditis uncommon; biomarker elevations more frequently observed with combination ICI regimens
Case-based and institutional observations [7]	Early ICI myocarditis cases; limited patient numbers	H-FABP occasionally elevated before or without hs-cTn increase	Troponin elevation generally occurred later during clinically overt myocarditis
Indirect inflammatory/immunometabolic evidence [24,31]	Patients with systemic inflammatory irAEs and elevated cytokine burden	H-FABP elevations associated with IL-6/CRP increase and oxidative-inflammatory activation	No overt cardiotoxicity in most cases; findings support relationship between immune activation and myocardial stress

**Table 4:** Ongoing clinical studies and registries informing biomarker-guided strategies for early detection and management of immune checkpoint inhibitor-associated cardiotoxicity.

Trial	Design & Population	Biomarkers	Primary Objectives & Endpoints	Relevance for H-FABP Integration
STRICT Trial (NCT06337097)	Prospective, interventional study in patients initiating immune checkpoint inhibitors	Structured troponin-guided surveillance (hs-cTns) during ICI therapy	Evaluate whether troponin-based monitoring improves early detection of myocardial injury and clinical outcomes	Establishes a biomarker-driven framework where fast-kinetic markers like H-FABP could be layered onto troponin to enhance temporal sensitivity
Abatacept for ICI Myocarditis (NCT05195645)	Phase II trial in patients with established ICI-associated myocarditis	Abatacept (CTLA-4 agonist) with mechanistic immune profiling	Assess immunosuppression efficacy, safety, and biomarker changes; adjudicated myocarditis outcomes	Provides a platform for testing H-FABP response to immunosuppressive therapy and correlation with troponin and MRI recovery
ICI-Myocarditis Registry (NCT04294771)	Multicenter observational registry of myocarditis cases across cancer types and ICI regimens	Troponin, BNP/NT-proBNP, ECG, CMR, immune profiling	Characterize phenotype, outcomes, diagnostic pathways; harmonize definitions	Opportunity to embed H-FABP as an exploratory biomarker benchmarked against CMR and clinical adjudication
Genomic Susceptibility Study (NCT06734689)	Genetic association study in patients receiving ICIs	Genomic risk loci, immune polymorphisms, cytokine signatures	Identify genetic predictors of ICI myocarditis	Can identify risk subgroups where adding an early marker such as H-FABP offers maximal predictive value
Biomarker-based Early Cardiac Injury Study (NCT06309862)	Observational biomarker-profiling study in ICI-treated cancer patients	Circulating biomarkers (multi-omic) for early myocardial injury	Detect early cardiotoxicity signals before clinical symptoms; correlation with imaging	Although H-FABP is not explicitly listed, study structure is ideal for evaluating H-FABP in parallel with hs-cTns and inflammatory cytokines
Abatacept/Kinetics Trials Extension Platforms (Related to NCT05195645 & registries)	Mechanistic biomarker studies embedded within ICI myocarditis treatment trials	Cytokines (IL-6, IL-1β), troponin, NT-proBNP, immune cell phenotyping	Map immunologic recovery and biomarker kinetics during treatment	Potential for assessing whether H-FABP normalization precedes troponin decline, supporting its use as a dynamic treatment-response biomarker

**Table 5:** Search strategy used in MEDLINE, EMBASE, and Web of Science.

Database	Search String
MEDLINE	“immune checkpoint inhibitors AND myocarditis” OR “ICI cardiotoxicity AND biomarkers” OR “H-FABP AND myocardial injury” OR “heart-type fatty acid-binding protein AND cardiotoxicity” OR “troponin AND immune checkpoint inhibitors” OR “cardio-oncology AND biomarkers”
EMBASE	“immune checkpoint inhibitors AND cardiac toxicity” OR “ICI myocarditis AND biomarkers” OR “H-FABP AND cardiac injury” OR “fatty acid binding protein AND myocarditis” OR “immune-related adverse events AND heart”
Web of Science	“ICI cardiotoxicity AND myocarditis” OR “H-FABP AND early myocardial injury” OR “cardio-oncology AND biomarkers” OR “immune checkpoint blockade AND heart”

## Data Availability

No new data were created or analyzed in this study. Data sharing is not applicable to this article.

## Abbreviations

The following abbreviations are used in this manuscript:

ASC	Apoptosis-associated speck-like protein containing a CARD
ATP	Adenosine triphosphate
BNP	B-type natriuretic peptide
CCL2	C-C motif chemokine ligand 2 (monocyte chemoattractant protein-1)
CCR2	C-C chemokine receptor type 2
CD3	Cluster of differentiation 3
CD8	Cluster of differentiation 8
CD28	Cluster of differentiation 28
CD36	Cluster of differentiation 36 (fatty acid translocase)
CD68	Cluster of differentiation 68
CK-MB	Creatine kinase MB isoenzyme
CMR	Cardiovascular magnetic resonance
CRP	C-reactive protein
CTLA-4	Cytotoxic T-lymphocyte-associated protein 4
CV-irAEs	Cardiovascular immune-related adverse events
DAMPs	Damage-associated molecular patterns
ECG	Electrocardiogram
EHA	European Hematology Association
ESC	European Society of Cardiology
ESTRO	European Society for Therapeutic Radiology and Oncology
FAO	Fatty-acid oxidation
H-FABP	Heart-type fatty acid-binding protein
hs-cTn	High-sensitivity cardiac troponin
hs-cTnI/T	High-sensitivity cardiac troponin I/T
ICI	Immune checkpoint inhibitor
ICIs	Immune checkpoint inhibitors
IC-OS	International Cardio-Oncology Society
IFN-γ	Interferon gamma
IL-1β	Interleukin-1 beta
IL-6	Interleukin-6
IL-8	Interleukin-8
IL-18	Interleukin-18
irAEs	Immune-related adverse events
JAK	Janus kinase
MACEs	Major adverse cardiovascular events
MPO	Myeloperoxidase
MyD88	Myeloid differentiation primary response 88
NF-κB	Nuclear factor kappa-light-chain-enhancer of activated B cells
NK	Natural killer (cell)
NLRP3	NOD-, LRR- and pyrin domain-containing protein 3 (inflammasome)
NT-proBNP	N-terminal pro–B-type natriuretic peptide
OX40	OX40 (CD134) co-stimulatory receptor
PD-1	Programmed cell death protein 1
PD-L1	Programmed cell death ligand 1
PI3K	Phosphoinositide 3-kinase
PPAR-α	Peroxisome proliferator-activated receptor alpha
RNS	Reactive nitrogen species
ROS	Reactive oxygen species
SDF-1	Stromal cell-derived factor 1
STAT3	Signal transducer and activator of transcription 3
TCR	T-cell receptor
TNF-α	Tumor necrosis factor alpha

## References

[1] Wang Y., Ding Q., Wei J. (2025). Current Advances and Future Directions of Combined ICIs and TILs in Solid Tumors. Cancer Lett..

[2] Chen Z., Song Z., Den S., Zhang W., Han M., Lan T., Du X., Ning J., Chen X., Lin H. (2025). Application of Immune Checkpoint Inhibitors in Cancer. MedComm.

[3] De Perna M.L., Rigamonti E., Zannoni R., Espeli V., Moschovitis G. (2025). Immune Checkpoint Inhibitors and Cardiovascular Adverse Events. ESC Heart Fail..

[4] Wang Y., Guo Y., Tan A.C., Zhao L., Shi X., Chen Y., Sun R.C., Liu M., Su J., George T.J. (2025). A real-world cohort study of immune-related adverse events in patients receiving immune checkpoint inhibitors. npj Precis. Oncol..

[5] Badaan S., Abbass A., Tov S., Bar-Sela G., Bisharat N. (2025). How should immune checkpoint inhibitor myocarditis be treated?. Cardiooncology.

[6] Salem J.E., Manouchehri A., Moey M., Lebrun-Vignes B., Bastarache L., Pariente A., Gobert A., Spano J.P., Balko J.M., Bonaca M.P. (2018). Cardiovascular toxicities associated with immune checkpoint inhibitors: An observational, retrospective, pharmacovigilance study. Lancet Oncol..

[7] Liu S., Chan J., Brinc D., Gandhi S., Izenberg A., Delgado D., Abdel-Qadir H., Wintersperger B.J., Thavendiranathan P. (2020). Immune Checkpoint Inhibitor-Associated Myocarditis with Persistent Troponin Elevation Despite Abatacept and Prolonged Immunosuppression. JACC CardioOncol..

[8] Dolladille C., Akroun J., Morice P.M., Dompmartin A., Ezine E., Sassier M., Da-Silva A., Plane A.F., Legallois D., L’Orphelin J.M. (2021). Cardiovascular immunotoxicities associated with immune checkpoint inhibitors: A safety meta-analysis. Eur. Heart J..

[9] Gruson D., Adamantidou C., Ahn S.A., Rousseau M.F. (2021). Heart-type fatty acid binding protein is related to severity and established cardiac biomarkers of heart failure. Adv. Lab. Med..

[10] Hotamisligil G.S., Bernlohr D.A. (2015). Metabolic functions of FABPs—Mechanisms and therapeutic implications. Nat. Rev. Endocrinol..

[11] Obaseki E., Adebayo D., Bandyopadhyay S., Hariri H. (2024). Lipid droplets and fatty acid-induced lipotoxicity: In a nutshell. FEBS Lett..

[12] Colli A., Josa M., Pomar J.L., Mestres C.A., Gherli T. (2007). Heart fatty acid binding protein in the diagnosis of myocardial infarction: Where do we stand today?. Cardiology.

[13] Goel H., Melot J., Krinock M.D., Kumar A., Nadar S.K., Lip G.Y.H. (2020). Heart-type fatty acid-binding protein: An overlooked cardiac biomarker. Ann. Med..

[14] Pavel A.L., Kundnani N.R., Morariu S.I., Tudor A., Man D.E., Duda-Seiman D.M., Velimirovici D.E., Valcovici M.D., Calin P., Dragan S.R. (2024). Importance of H-FABP in Early Diagnosis of Acute Myocardial Infarction. Int. J. Gen. Med..

[15] Mad P., Domanovits H., Fazelnia C., Stiassny K., Russmüller G., Cseh A., Sodeck G., Binder T., Christ G., Szekeres T. (2007). Human heart-type fatty-acid-binding protein as a point-of-care test in the early diagnosis of acute myocardial infarction. QJM.

[16] Kilcullen N., Viswanathan K., Das R., Morrell C., Farrin A., Barth J.H., Hall A.S., EMMACE-2 Investigators (2007). Heart-type fatty acid-binding protein predicts long-term mortality after acute coronary syndrome and identifies high-risk patients across the range of troponin values. J. Am. Coll. Cardiol..

[17] Leven A.S., Wagner N., Nienaber S., Messiha D., Tasdogan A., Ugurel S. (2025). Changes in tumor and cardiac metabolism upon immune checkpoint. Basic. Res. Cardiol..

[18] Sebastián D., Guitart M., García-Martínez C., Mauvezin C., Orellana-Gavaldà J.M., Serra D., Gómez-Foix A.M., Hegardt F.G., Asins G. (2009). Novel role of FATP1 in mitochondrial fatty acid oxidation in skeletal muscle cells. J. Lipid Res..

[19] Houten S.M., Wanders R.J. (2010). A general introduction to the biochemistry of mitochondrial fatty acid β-oxidation. J. Inherit. Metab. Dis..

[20] Vupputuri A., Sekhar S., Krishnan S., Venugopal K., Natarajan K.U. (2015). Heart-type fatty acid-binding protein (H-FABP) as an early diagnostic biomarker in patients with acute chest pain. Indian Heart J..

[21] Park J., Skålhegg B.S. (2025). Combination of PD-1/PD-L1 and CTLA-4 inhibitors in the treatment of cancer—A brief update. Front. Immunol..

[22] Adel F.W., Saleh G., Anavekar N.S., Hayes S.N. (2025). Delayed-Onset ICI-Associated Myocarditis: When Immunotherapy’s Cardiac Consequences Outlast Treatment. JACC Case Rep..

[23] Yao G., Zhang Y., Zhang H., Tang L., Ding C., Zhou X. (2025). Immune checkpoint inhibitor-associated myocarditis and pericarditis: A pharmacovigilance study based on the FAERS database. BMC Cancer.

[24] Miteva K., Anker M.S., Fechner H., Lehmann L., Van Linthout S. (2025). Cancer-Related Immune Therapies: Bidirectional Implications From Cardiotoxicity to Emerging Cardiovascular Therapeutics: JACC CardioOncology State-of-the-Art Review. JACC CardioOncol..

[25] Quagliariello V., Passariello M., Di Mauro A., Cipullo C., Paccone A., Barbieri A., Palma G., Luciano A., Buccolo S., Bisceglia I. (2022). Immune checkpoint inhibitor therapy increases systemic SDF-1, cardiac DAMPs Fibronectin-EDA, S100/Calgranulin, galectine-3, and NLRP3-MyD88-chemokine pathways. Front. Cardiovasc. Med..

[26] Quagliariello V., Passariello M., Rea D., Barbieri A., Iovine M., Bonelli A., Caronna A., Botti G., De Lorenzo C., Maurea N. (2020). Evidences of CTLA-4 and PD-1 Blocking Agents-Induced Cardiotoxicity in Cellular and Preclinical Models. J. Pers. Med..

[27] Cortellino S., Quagliariello V., Delfanti G., Blaževitš O., Chiodoni C., Maurea N., Di Mauro A., Tatangelo F., Pisati F., Shmahala A. (2023). Fasting mimicking diet in mice delays cancer growth and reduces immunotherapy-associated cardiovascular and systemic side effects. Nat. Commun..

[28] D’Anneo A., Bavisotto C.C., Gammazza A.M., Paladino L., Carlisi D., Cappello F., de Macario E.C., Macario A.J.L., Lauricella M. (2020). Lipid chaperones and associated diseases: A group of chaperonopathies defining a new nosological entity with implications for medical research and practice. Cell Stress. Chaperones.

[29] Huang H., McIntosh A.L., Martin G.G., Petrescu A.D., Landrock K.K., Landrock D., Kier A.B., Schroeder F. (2013). Inhibitors of Fatty Acid Synthesis Induce PPAR α-Regulated Fatty Acid β-Oxidative Genes: Synergistic Roles of L-FABP and Glucose. PPAR Res..

[30] Quagliariello V., Passariello M., Bisceglia I., Paccone A., Inno A., Maurea C., Rapuano Lembo R., Manna L., Iovine M., Canale M.L. (2024). Combinatorial immune checkpoint blockade increases myocardial expression of NLRP-3 and secretion of H-FABP, NT-Pro-BNP, interleukin-1β and interleukin-6: Biochemical implications in cardio-immuno-oncology. Front. Cardiovasc. Med..

[31] Zhou F., Liu G., Zhang S., Luo C., Hu S., Wan S., Xiong W., Zhao L. (2025). Cardiotoxicity in cancer immunotherapy: A systematic review and global meta-analysis. J. Transl. Med..

[32] Gergely T.G., Drobni Z.D., Sayour N.V., Ferdinandy P., Varga Z.V. (2025). Molecular fingerprints of cardiovascular toxicities of immune checkpoint inhibitors. Basic. Res. Cardiol..

[33] Jensen G., Wang X., Kuempel J., Chen Z., Yu W., Palaskas N., Sobieski M., Nguyen N., Powell R.T., Stephan C. (2024). Modeling immune checkpoint inhibitor associated myocarditis in vitro and its therapeutic implications. J. Mol. Cell. Cardiol. Plus.

[34] Won T., Kalinoski H.M., Wood M.K., Hughes D.M., Jaime C.M., Delgado P., Talor M.V., Lasrado N., Reddy J., Čiháková D. (2022). Cardiac myosin-specific autoimmune T cells contribute to immune-checkpoint-inhibitor-associated myocarditis. Cell Rep..

[35] Ghafouri-Fard S., Khanbabapour Sasi A., Hussen B.M., Shoorei H., Siddiq A., Taheri M., Ayatollahi S.A. (2022). Interplay between PI3K/AKT pathway and heart disorders. Mol. Biol. Rep..

[36] Nef H.M., Möllmann H., Hilpert P., Troidl C., Voss S., Rolf A., Behrens C.B., Weber M., Hamm C.W., Elsässer A. (2009). Activated cell survival cascade protects cardiomyocytes from cell death in Tako-Tsubo cardiomyopathy. Eur. J. Heart Fail..

[37] Chin C.G., Chen Y.C., Lin F.J., Lin Y.K., Lu Y.Y., Cheng T.Y., Chen S.A., Chen Y.J. (2024). Targeting NLRP3 signaling reduces myocarditis-induced arrhythmogenesis and cardiac remodeling. J. Biomed. Sci..

[38] Napodano C., Carnazzo V., Basile V., Pocino K., Stefanile A., Gallucci S., Natali P., Basile U., Marino M. (2023). NLRP3 Inflammasome Involvement in Heart, Liver, and Lung Diseases-A Lesson from Cytokine Storm Syndrome. Int. J. Mol. Sci..

[39] Wang X., He J., Ding G., Tang Y., Wang Q. (2025). Overcoming resistance to PD-1 and CTLA-4 blockade mechanisms and therapeutic strategies. Front. Immunol..

[40] Zeng Z., You M., Fan C., Jang J., Xia X. (2025). FABP5 regulates ROS-NLRP3 inflammasome in glutamate-induced retinal excitotoxic glaucomatous model. FASEB J..

[41] Floy M.E., Dunn K.K., Mateyka T.D., Reichardt I.M., Steinberg A.B., Palecek S.P. (2022). Direct coculture of human pluripotent stem cell-derived cardiac progenitor cells with epicardial cells induces cardiomyocyte proliferation and reduces sarcomere organization. J. Mol. Cell. Cardiol..

[42] Carless D.R., Wnęk M., Knox C., Harrison K.R., Calder N., Hall A.S., Barth J.H. (2013). Clinical and analytical evaluation of an immunoturbidimetric heart-type fatty acid-binding protein assay. Scand. J. Clin. Lab. Invest..

[43] Kim Y., Kim H., Kim S.Y., Lee H.K., Kwon H.J., Kim Y.G., Lee J., Kim H.M., So B.H. (2010). Automated heart-type fatty acid-binding protein assay for the early diagnosis of acute myocardial infarction. Am. J. Clin. Pathol..

[44] Gururajan P., Gurumurthy P., Nayar P., Srinivasa Nageswara Rao G., Babu S., Cherian K.M. (2010). Heart fatty acid binding protein (H-FABP) as a diagnostic biomarker in patients with acute coronary syndrome. Heart Lung Circ..

[45] Nayashida N., Chihara S., Tayama E., Akasu K., Kai E., Kawara T., Aoyagi S. (2001). Influence of renal function on serum and urinary heart fatty acid-binding protein levels. J. Cardiovasc. Surg..

[46] Rezar R., Jirak P., Gschwandtner M., Derler R., Felder T.K., Haslinger M., Kopp K., Seelmaier C., Granitz C., Hoppe U.C. (2020). Heart-Type Fatty Acid-Binding Protein (H-FABP) and its Role as a Biomarker in Heart Failure: What Do We Know So Far?. J. Clin. Med..

[47] Yuan M., Zang L., Xu A., Gong M., Liu Q., Huo B., Wang J., Fu H., Tse G., Roever L. (2021). Dynamic Changes of Serum Heart Type-Fatty Acid Binding Protein in Cancer Patients Treated with Immune Checkpoint Inhibitors. Front. Pharmacol..

[48] Tonomura Y., Matsushima S., Kashiwagi E., Fujisawa K., Takagi S., Nishimura Y., Fukushima R., Torii M., Matsubara M. (2012). Biomarker panel of cardiac and skeletal muscle troponins, fatty acid binding protein 3 and myosin light chain 3 for the accurate diagnosis of cardiotoxicity and musculoskeletal toxicity in rats. Toxicology.

[49] Thygesen K., Alpert J.S., Jaffe A.S., Chaitman B.R., Bax J.J., Morrow D.A., White H.D., Executive Group on behalf of the Joint European Society of Cardiology (ESC), American College of Cardiology (ACC), American Heart Association (AHA) (2018). Fourth Universal Definition of Myocardial Infarction (2018). Circulation.

[50] Alhadi H.A., Fox K.A. (2010). Heart-Type Fatty Acid-Binding Protein in the Early Diagnosis of Acute Myocardial Infarction: The potential for influencing patient management. Sultan Qaboos Univ. Med. J..

[51] Pelsers M.M., Hermens W.T., Glatz J.F. (2005). Fatty acid-binding proteins as plasma markers of tissue injury. Clin. Chim. Acta.

[52] Nakata T., Hashimoto A., Hase M., Tsuchihashi K., Shimamoto K. (2003). Human heart-type fatty acid-binding protein as an early diagnostic and prognostic marker in acute coronary syndrome. Cardiology.

[53] Xu J., Zhou Z., Zheng Y., Yang S., Huang K., Li H. (2023). Roles of inflammasomes in viral myocarditis. Front. Cell Infect. Microbiol..

[54] Herrmann J., Barac A., Carver J., Cheng R.K., Daniele A., Dent S., Deych E., Lee D.H., Lenihan D., Leong D.P. (2025). Immune Checkpoint Inhibitor-Associated Cardiovascular Toxic Effects: International Cardio-Oncology Society Position Statement. JAMA Oncol..

[55] Jo W., Sun V., Čiháková D. (2026). Immune Mechanisms of Viral, Autoimmune, and Immune Checkpoint Inhibitor-Associated Myocarditis. Immunol. Rev..

[56] Deharo F., Thuny F., Cadour F., Resseguier N., Meilhac A., Gaubert M., Dolladille C., Paganelli F., Alexandre J., Cautela J. (2023). Diagnostic Value of the International Society of Cardio-Oncology Definition for Suspected Immune Checkpoint Inhibitor-Associated Myocarditis. J. Am. Heart Assoc..

[57] Nardin S., Ruffilli B., Costantini P., Mollace R., Taglialatela I., Pagnesi M., Chiarito M., Soldato D., Cao D., Conte B. (2025). Navigating Cardiotoxicity in Immune Checkpoint Inhibitors: From Diagnosis to Long-Term Management. J. Cardiovasc. Dev. Dis..

[58] Cheng L., Xu Y., Zhang S. (2023). Cardiovascular and Oncological Outcomes in Immune Checkpoint Inhibitor-Induced Myocarditis: Balancing Perspectives. JACC CardioOncol..

[59] Kenyon C.R., Van Wyk L., Flom A., Ibrahim R., Nhat Pham H., Lakhdar S., Iftikhar M., Abdelnabi M. (2026). Advances in Diagnosis and Treatment of Acute and Chronic Heart Failure: A Comprehensive Review. J. Clin. Med..

[60] Wang C., Zhao G., Zhang Z., Yang L., Liu S., Li G., Wang H., Huang J., Wang S., Li N. (2023). Immune checkpoint inhibitor-associated myocarditis: A systematic analysis of case reports. Front. Immunol..

[61] Zhang L., Awadalla M., Mahmood S.S., Nohria A., Hassan M.Z.O., Thuny F., Zlotoff D.A., Murphy S.P., Stone J.R., Golden D.L.A. (2020). Cardiovascular magnetic resonance in immune checkpoint inhibitor-associated myocarditis. Eur. Heart J..

[62] Lyon A.R., López-Fernández T., Couch L.S., Asteggiano R., Aznar M.C., Bergler-Klein J., Boriani G., Cardinale D., Cordoba R., Cosyns B. (2022). 2022 ESC Guidelines on cardio-oncology developed in collaboration with the European Hematology Association (EHA), the European Society for Therapeutic Radiology and Oncology (ESTRO) and the International Cardio-Oncology Society (IC-OS). Eur. Heart J..

[63] von Kemp B., Halvorsen S., Nohria A. (2022). The new 2022 ESC Guidelines on Cardio-oncology and their impact on the Acute Cardiovascular Care Society. Eur. Heart J. Acute Cardiovasc. Care.

[64] López-Fernández T., Lyon A.R., Herrmann J. (2022). 2022 ESC Guidelines on cardio-oncology: How can we improve the cardiovascular health of patients with cancer and cancer survivors?. Eur. Heart J. Cardiovasc. Pharmacother..

[65] Haltern G., Peiniger S., Bufe A., Reiss G., Gülker H., Scheffold T. (2010). Comparison of usefulness of heart-type fatty acid binding protein versus cardiac troponin T for diagnosis of acute myocardial infarction. Am. J. Cardiol..

[66] Zhang H.W., Jin J.L., Cao Y.X., Liu H.H., Zhang Y., Guo Y.L., Wu N.Q., Gao Y., Xu R.X., Hua Q. (2020). Prognostic utility of heart-type fatty acid-binding protein in patients with stable coronary artery disease and impaired glucose metabolism: A cohort study. Cardiovasc. Diabetol..

[67] Zamzam A., Syed M.H., Rotstein O.D., Eikelboom J., Klein D.J., Singh K.K., Abdin R., Qadura M. (2022). Validating fatty acid binding protein 3 as a diagnostic and prognostic biomarker for peripheral arterial disease: A three-year prospective follow-up study. eClinicalMedicine.

[68] STRICT—Surveillance with TRoponin During Immune Checkpoint Therapy. https://clinicaltrials.gov/study/NCT06337097.

[69] Salem J.E., Ederhy S., Belin L., Zahr N., Tubach F., Procureur A., Allenbach Y., Rosenzwjag M., Bretagne M. (2025). Abatacept dose-finding phase II triaL for immune checkpoint inhibitors myocarditis (ACHLYS) trial design. Arch. Cardiovasc. Dis..

[70] Power J.R., Dolladille C., Ozbay B., Procureur A.M., Ederhy S., Palaskas N.L., Lehmann L.H., Cautela J., Courand P.Y., Hayek S.S. (2024). Predictors and Risk Score for Immune Checkpoint-Inhibitor-Associated Myocarditis Severity. medRxiv.

[71] Genetic Determinants of Myocarditis Induced by Immune-Checkpoint Inhibitors (GICICA-Control). https://clinicaltrials.gov/study/NCT06734689.

[72] Immune Checkpoint Inhibitor Therapy for Cancer and Risk of Myocarditis or Cardiomyopathy. https://clinicaltrials.gov/study/NCT06309862.

